# Compendious survey of protein tandem repeats in inbred mouse strains

**DOI:** 10.1186/s12863-022-01079-1

**Published:** 2022-08-05

**Authors:** Ahmed Arslan

**Affiliations:** 1 Stanford University School of Medicine, 300 Pasteur Drive, Palo Alto, CA 94504 USA; 2 Present address: Sanford Burnham Prebys Medical Discovery Institute, 10901 N Torrey Pines Rd, La Jolla, CA 92037 USA

**Keywords:** Short tandem repeats (STRs), Alleles, Mouse, Phenotype, Protein, 3-dimensional models, Protein structure

## Abstract

Short tandem repeats (STRs) play a crucial role in genetic diseases. However, classic disease models such as inbred mice lack such genome wide data in public domain. The examination of STR alleles present in the protein coding regions (are known as protein tandem repeats or PTR) can provide additional functional layer of phenotype regulars. Motivated with this, we analysed the whole genome sequencing data from 71 different mouse strains and identified STR alleles present within the coding regions of 562 genes. Taking advantage of recently formulated protein models, we also showed that the presence of these alleles within protein 3-dimensional space, could impact the protein folding. Overall, we identified novel alleles from a large number of mouse strains and demonstrated that these alleles are of interest considering protein structure integrity and functionality within the mouse genomes. We conclude that PTR alleles have potential to influence protein functions through impacting protein structural folding and integrity.

**Supplementary Information:**

The online version contains supplementary material available at 10.1186/s12863-022-01079-1.

## Introduction

Short tandem repeats (STRs) or microsatellites consist of 1—6 base-pair long consecutively repeating units and represent a major source of genetic variability [1]. It has been shown that STRs compose about 1% of the human genome and regulate genes. Moreover, STRs contribute to more than 30 mendelian disorders as well as complex traits [1]. The abnormal extension of protein coding regions (PTRs) could result in longer polypeptides compared to wildtype and that may lead to abnormal protein interactions [2]. PolyQ diseases are a group of neurodegenerative disorders, resulting from CAG repeats present within the protein coding regions that could alter protein conformation and trigger loss-of-function effects by disrupting normal protein functions [3].

In comparison to the traditional PCR-based STRs detection methods, recent advances in genomic platform and algorithm development made way for the whole genome based STRs detection. Several methods have been developed to sample STR alleles from whole genome sequencing data [4]. These efforts have led to the understanding of the function of STRs in healthy and diseased human samples as well as in model organisms [5]. Among lab models, mice are one of the primer model organisms to study human diseases [6]. The possibility of producing genetically modified animals, of relatively small size, and within a small gestation period make mice models ideal to study effects of genetic variations [7]. Several decades of research have made this an ideal specimen to understand the role of genetic variations and interpret the impact of these aberrations with respect to biomedical traits [7]. Although genetic variations like single nucleotide polymorphism (SNPs) [6] and structural variants (SVs) [8] from a large number of mice strains have been reported, that isn’t the case for STRs. We argue that STR allele sampling could be an important step towards the proper understanding of protein functions within individual strains, in addition to SNPs and indels.

Considering the importance of mouse models to study human diseases, such as neurodevelopmental diseases like autism, it is crucial to delineate completely the underlying genetics. Autism spectrum disease (ASD) is a collection of neurological disorders that affects the way subjects communicate and behave [9]. According to CDC, the number of patients per year for ASD are increasing [10]. The complex disease genetics are still not completely understood. Recent studies on human autistic patients have shown that they carry STR regions, which suggests the importance and relevance of studying these regions to gain a better understanding of the disease [5]. We recently showed that autism mouse model has a unique genetic makeup causing abnormal neuroanatomy, that could impact its social behaviour [8]. For this model and others, the complete genetic map of STRs, especially those present within coding regions (PTR), is still lacking. Given the importance of STRs, it crucial to identify these alleles from mouse genome and suggest their potential impact on protein functions.

Therefore, in this study we identify the PTR alleles from mouse genome(s) and suggest the functional importance of these alleles. Moreover, we use a computational framework to assess the distortion impact of PTRs on the protein folding by integrating repeats to molecular dynamics data. Our results suggest that the PTR alleles could impact protein structure and have potential to change protein function too.

## Results

**Fig. 1:**
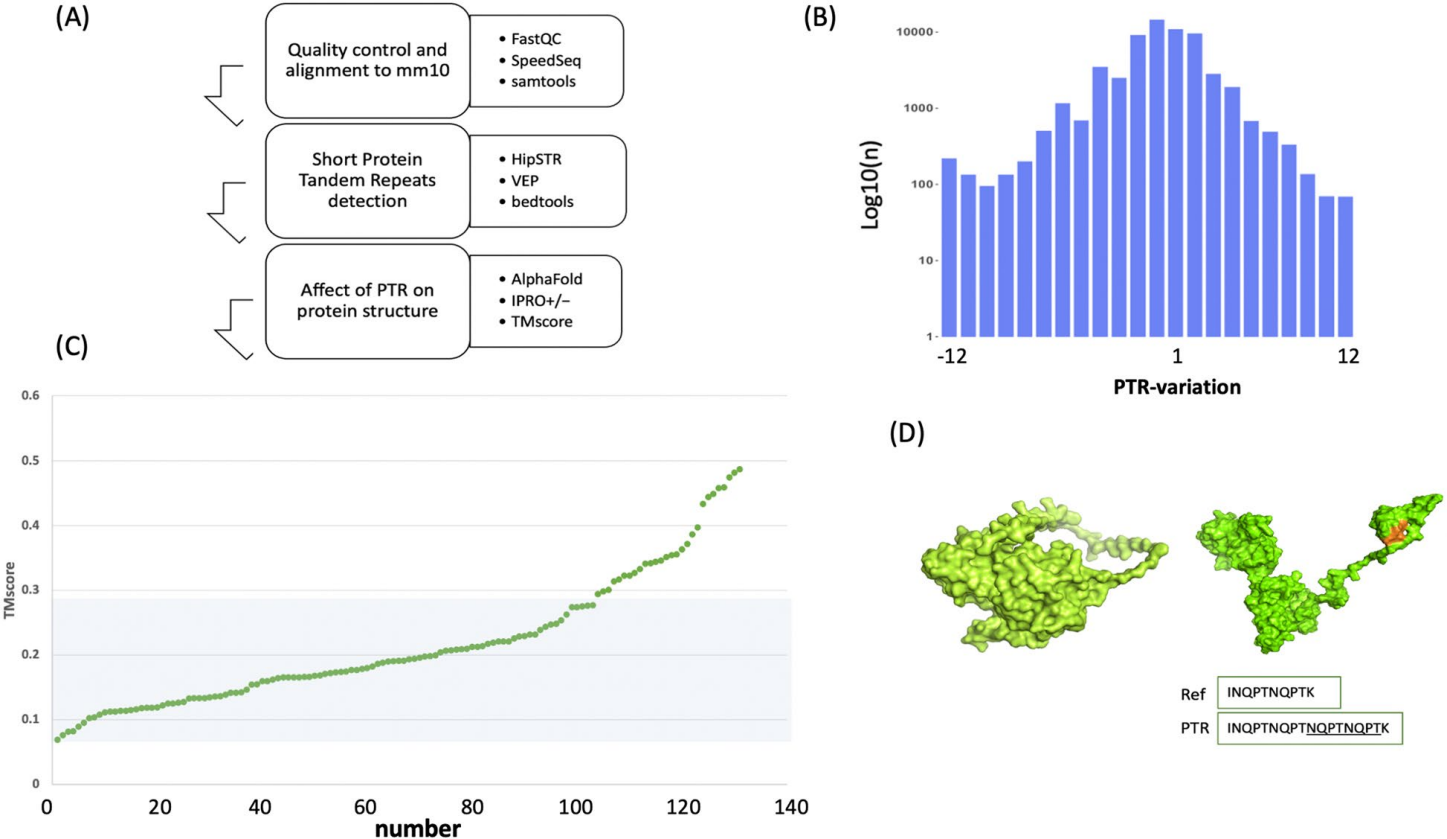
Identification of PTR (**A**) analysis steps performed, from sequence alignment to PTR detection to assessment of potential impact of tandem repeats present in the protein structures, are shown. **B** PTR allele variations with numbers of each variant are shown. Horizontal axis shows the allele type, positive = expansion; negative = contraction whereas vertical axis shows the number (log10-transformation). **C** number of PTR alleles are plotted against their TMscore, darker horizontal bar shows the number of alleles with score less than 0.3. **D** Assessment of PTR alleles impact of *Sirt3* protein model, right, predicted protein model, left, protein folding upon the presence of PTR allele NQPTNQPT (shown in brown color and underlined in the sequence box below). Alternative folding of templates (TMscore = 0.24) is impacted by the PTR allele present in 58 strains. Two boxes below show the reference allele and PTR allele motif

To understand the function of protein tandem repeats in inbred mice, we collected whole genome sequencing data for 71 strains with a mean read depth of 39.5 × from sequence reads archive (SRA) (Table S1). The repeats were identified with the HipSTR algorithm [1] and a stringent cut-off read depth criteria of 25 × was used to produce robust results (see details in material and methods) (Fig. 1A). This framework identified 941 PTR variable alleles in 562 protein coding genes from our samples, which makes on average ~ 14 alleles per strain (Table S2). We observed little differences in the distribution of PTR alleles between N-terminus (25%) and C-terminus (32%) of polypeptides. We also identified a group of 165 proteins which contains PTR alleles but no SNP or indel alleles (Table S3). The list includes many important genes including homeobox genes important regulators of crucial functions (see discussion for details). We also observed variable PTR allele length distribution in the range of ± 12 amino-acids in comparison to reference (Fig. 1B). With our computational dynamics approach we also observed that the protein folding was impacted by the presence of PTRs (see below).

We detected 120 PTR alleles overlapping 88 different types of protein domains from 92 proteins (Fig S1, Table S4). The domain type with the most overlapping PTR alleles (*n* = 21) is RNA recognition motif (RRM). Interestingly, we identified two PTR alleles present inside the homeobox domain of *Dlx6* and *Esx1* proteins. Overall, these PTR alleles can impact the evolutionary conserved functions of mouse protein domains.

We then investigated whether the presence of PTR could impact the protein structural stability or template folding. More specifically, the presence of PTR allele could create alternative residue spacing in 3-dimensional polypeptide backbone that could, in return, lead to novel protein interaction accessibility and/or functions. To test this hypothesis, we simulated the PTR alleles within protein models by applying a method (IPRO ±) specialising in detecting molecular dynamic changes upon the presence of the alternative alleles inside protein models [4]. We applied this method to more than 180 protein models available for the PTR alleles carrying proteins, retrieved from the AlphaFold protein structure database [11]. To quantify the changes, we compared AlphaFold models without PTR alleles to the PTR-containing models by aligning two protein models with the TMalign algorithm. In models comparison, 131 cases show a TMscore of less than 0.5, and 105 cases with a TMscore of less than 0.3 (Fig. 1C). A score ranging from 0.1–0.3 shows that two aligned structures have random structural similarity [12]. Out of 131 cases with a TMscore under 0.5, 24 PTR alleles are present within the protein functional domains (n = 52). This observation suggests that impactful PTR alleles are present outside functional domains. Our computational dynamic results indicate that the presence of PTR alleles impacts protein folding prospects, which could deviate protein interaction and functions (Fig. 1D).

The characterization of composition of PTR alleles producing lowest TMscore(s) can bring more insights on the nature and composition of these alleles. We observed a weak correlation between the length of the PTR alleles and the observed TMscore values of PTRs (Pearson’s cor. test, *p*-value = 0.60). We, then, trained a multiple regression model to predict the impact of predictor variables such as allele length, position (i.e., N- or C-terminus), type of allele (i.e., extension or contraction) and collective mass of amino acids constituting a PTR allele on the TMscore. In this analysis, we observed a strong statistically significant association between the type of PTR allele and TMscore (*p*-value = 9.39e-06). However, no associations of length and collective amino-acid mass to the TMscore were observed. Within a given PTR allele type, the mass of extension allele is significantly associated with TMscore (*p*-value = 0.009) whereas PTR length has a weak association with TMscore (*p*-value = 0.02). This shows that contraction or extension of the PTR allele could have profound impact on the protein folding compared to the length of the PTR allele or other variables such as collective mass of amino acids present within a PTR allele.

Next, we analysed a set of genes (*n* = 2609) known to play a role in neurodevelopmental disorders including autism. The aim was to identify PTR alleles from these genes and to suggest that these disease regulators carry new types of polymorphisms. We identified 164 unique PTR alleles present in 92 genes from this set of genes (Table S5). Although most of these alleles are common, we also identified two rare alleles (MAF < 0.05) that belong to two different genes, *Gigyf2* and *Hectd4*. Both genes are high confidence autism associated genes and both have an extension of one amino acid (Q and A, respectively) in five difference strains (129S1, BTBR, FVB, RHJ and WSB). The 129S1 and BTBR strains are well established autism models. Several studies have shown genetic, transcriptomic and proteomics variability present in these models especially in BTBR [13–15], however, the PTR alleles present in these genes not been reported previously. To our knowledge, this study is the first to identify the presence of PTR alleles within autism associated genes from several mouse strains. These previously unknown PTR alleles present within the ASD-related genes from mouse genomes could offer new insights into disease regulation mechanisms from mouse models such as BTBR.

## Material and methods

We analysed whole genome sequencing data from 71 different inbred mouse strains and identified STRs present in the protein coding region or PTRs. We retrieved raw whole genome sequencing data (fastq file format) of inbred mouse strains from the Sequence Read Archive (SRA). An initial quality control was performed with fastqc [16] and quality reads were aligned to reference mm10 genome with SpeedSeq pipeline, *speedseq align* parameter [17]. The output of alignment was sorted in a binary alignment map (bam) file format with samtools [18]. Tandem repeats were identified using the HipSTR pipeline [1] with minimum reads support for an STR allele set to 25 reads (parameter: *–min-reads 25*). Briefly, HipSTR, the STR detection started with the learning stutter noise profile from the input data (parameter: *–def-stutter-model*). Then, for genomic location of repeats it utilized the profile from the previous step and realigned STR-containing reads to guess haplotype information by using the hidden Markov model (HMM). The strategy reduced PCR stutter effects present in the input reads. The realignment was a crucial step in the framework to produce most likely STR alleles, and to perform accurate allele genotyping [1]. The final output of HipSTR is a variant call file (vcf) format. After filtering as recommended *(–min-call-qual 0.9 –max-call-flank-indel 0.15 –max-call-stutter 0.15*) [1] we selected homozygous alleles with the *bedtools query* command to proceed further. We then performed the genomic annotation with the Ensembl variant effect predictor (VEP) tool for mm10 (v100)[19]. The output files from the annotation step were further filtered for the annotations predicted as “protein altering variant”.

We retrieved protein models from the AlphaFold database [20] for the proteins that contain PTRs. For each protein model, we introduced an addition or deletion of a PTR allele within the model and assessed the effects of this edition with a pyrosetta-based framework, called IPRO ± [21]. Briefly, the IPRO ± approach spreads over several steps: calculation of sequence alignment driven probability statistics for substitutions, polypeptide backbone propagation for the indels, rotamer repackaging, target molecule containing indels repackaging, energy minimization, template refinement and interaction energy calculation, and reiterations until the production of a stable model. For complete information of the algorithm, see [21]. The resulting protein models from the IPRO ± approach were compared to the models without PTR alleles (to assess the impact of alleles) by aligning two models with TMalign algorithm [22]. In TMalign, the algorithm first generates structural alignment at residue level by applying heuristic dynamic programming iterations and this alignment is used to generate optimal superposition of the two structures. In the end, the method returns a template modelling score (TMscore) to show the extent of match between two models. A TMscore < 0.3 shows a randomness of the structure similarly and TMscore > 0.5 denotes the protein folds are same [22].

For the multiple regression model, we fit the data with the given equation:

1 $$\upgamma (\mathrm{tms}) = {\upbeta }_{0} + {\upbeta }_{1(\mathrm{len})} + {\upbeta }_{2(\mathrm{mass})} + {\upbeta }_{3(\mathrm{type})} +\upvarepsilon$$

where γ _(tms)_ is TMscore, β_0_ is intercept, and ε is error term, β_1(len)_, β_2(mass),_ β_3(type)_ are length, mass, and allele type variables, respectively. Equation (1) was used to predict the dependence of TMscore of protein models on the type of PTR allele, extension or deletion, mass of amino acids constituting an allele, or length of the allele. The model residue independence and normal distribution was analysed with the Durbin-Watson test and the Jarque Bera test, respectively. For both tests, a threshold of p-value < 0.05 was used to test the significance.

To compile a comprehensive set of disease-related genes, we collected up to date lists of neurodevelopmental disorder genes including autism associated genes from the SFAI genes database (https://gene.sfari.org/) and from a recent literature survey [23].

## Discussion

In this study, we aimed to identify the tandem repeats present inside the protein coding region from mouse genome, and to suggest potential functional features of PTR alleles. We findings suggested that (i) mouse proteins contain tandem repeats, (ii) PTR alleles can also be present inside the evolutionary conserved domains, (iii) protein folding properties can diverge from their wild-type state upon the presence of PTR alleles, and (iv) disease associated genes could also retain PTR alleles. Together, the novel mouse PTR datasets generated in this study suggested that these repeats could potentially impact protein functions by modulating protein stability and folding.

We previously have shown that the SNPs, indels and SVs can play a major role in mouse phenotypic variations [15, 24]. However, these and other studies focused on finding the association of genetic variations to mouse phenotypes lack power to fully explain phenotypic variations. This limitation could be diminished by analysing additional types of genetic variations such as PTRs. Here, we documented PTR alleles in 562 proteins from 71 mouse genomes, and their potential to contribute towards protein folding. Previous studies have established that the presence of even one additional amino acid can impact the function and stability of the protein [25]. Our results indicate that a large variation due to PTR alleles is present in the mouse proteins which could alter wildtype protein folding. We also observed, a set of 165 proteins that contain PTR alleles, but no SNP or indel alleles. This set included several crucial proteins such as homeobox factors, for example *Hoxa11, Hoxb3* and *Hoxd13*. This observation shows that a large group of repeat alleles were unnoticed previously and could contribute to deviating predictability of phenotypic variations.

Additionally, we have shown several crucial features of PTR alleles (as mentioned above). Recently reported homo, small and micro-repeats that are located at both N- and C-terminal [26], we also observed here,  the mouse PTRs were present in almost the same numbers at both terminals. Previous findings suggested that the most frequent PTR containing protein domains in eukaryotes include WD40, zf-C2H2, LRR_8 and RRM [26]. Our results suggested the RRM domain is the most frequent domain-type from our studied strains (Fig S1). The RRM domains are typically 90 amino-acid long and considered as the multifunctional regulators of development, cell differentiation, signalling, and gene expression [4]. In addition, PTRs present within homeobox domains were also identified. Homeobox domains regulate gene expression during the cell differentiation at early embryogenesis stages. Unsurprisingly, genetic anomalies in these regions cause developmental defects with severe consequences such as loss or deformation of body segments [27].

Perhaps the most interesting PTR feature is the detection of these alleles from disease associated proteins. Previous understanding about these disease related proteins was based on variations that are not PTR. This observation shows that a disease associated protein might not carry disease causing SNP/Indel/SV, but PTR allele(s). For instance, the rare extension PTR alleles present within the *Gigyf2* and *Hectd4* proteins, could have been left undetected if SNP or indel variations were the focus of a study to explain phenotypic variation. The inclusion of PTR alleles alongside with other type of alternative alleles can aid in providing a comprehensive map of mouse genomic variations. Future studies should take advantage of such datasets to perform more effective mouse genotype to phenotype association analysis. Together, the datasets produced in this study potentially facilitate depth of analyses to future studies identifying more broadly the phenotype regulatory factors.

The availability of highly accurate protein models from novel algorithms like AlphaFold made it feasible to analyse and produce reliable results. Moreover, new sequencing technologies such as long-read sequencing can further enhance analyses of genomic variations. As we relayed of short-read data which traditionally suffer limitation in identification of variations when length of an allele in under consideration. In this regard, our study might have limitations. Nevertheless, we are hoping that future studies will contribute to the identification of additional PTR alleles with the use of the above-mentioned technologies and add depth to the remaining missing links between phenotype and genotype.

In conclusion, we have shown that the PTR alleles from mouse genomes have several functional features, and that a better understanding of these alleles could help improve the apprehension of outcomes from mouse phenotype-based experiments. We showed that (i) the PTR alleles are present within functional protein regions and domains, (ii) they potentially can impact protein folding, (iii) and that disease associated genes also carry PTR alleles. With this study, we contribute to further establishing the importance of protein repeat regions in the mouse genome and to stressing the need to include repeat alleles in future studies.

## Supplementary Information

**Additional file 1: Fig S1.** PTR extension alleles inside protein domains.

**Additional file 2: Table S1.** Whole genome sequencing data from inbred mouse strains analysed in this study. **Table S2.**PTR alleles identified in the study. **TableS3.** Proteins with PTR allele with no SNP or Indel alleles. **Table S4.** Protein domains with PTR alleles. **Table S5.** PTR present within the neurodevelopmental disorders associated genes.

## Data Availability

The datasets analysed during the current study are publicly available in the Sequence Read Archive (SRA) repository, the accession numbers of each dataset are provided in the Table-S1.
