# Supplementary figures and images for: Compendious survey of protein tandem repeats in inbred mouse strains
Source: BMC Genom Data. 2022 Aug 5;23:62. doi: 10.1186/s12863-022-01079-1 (PMC9354378; doi:10.1186/s12863-022-01079-1)

**
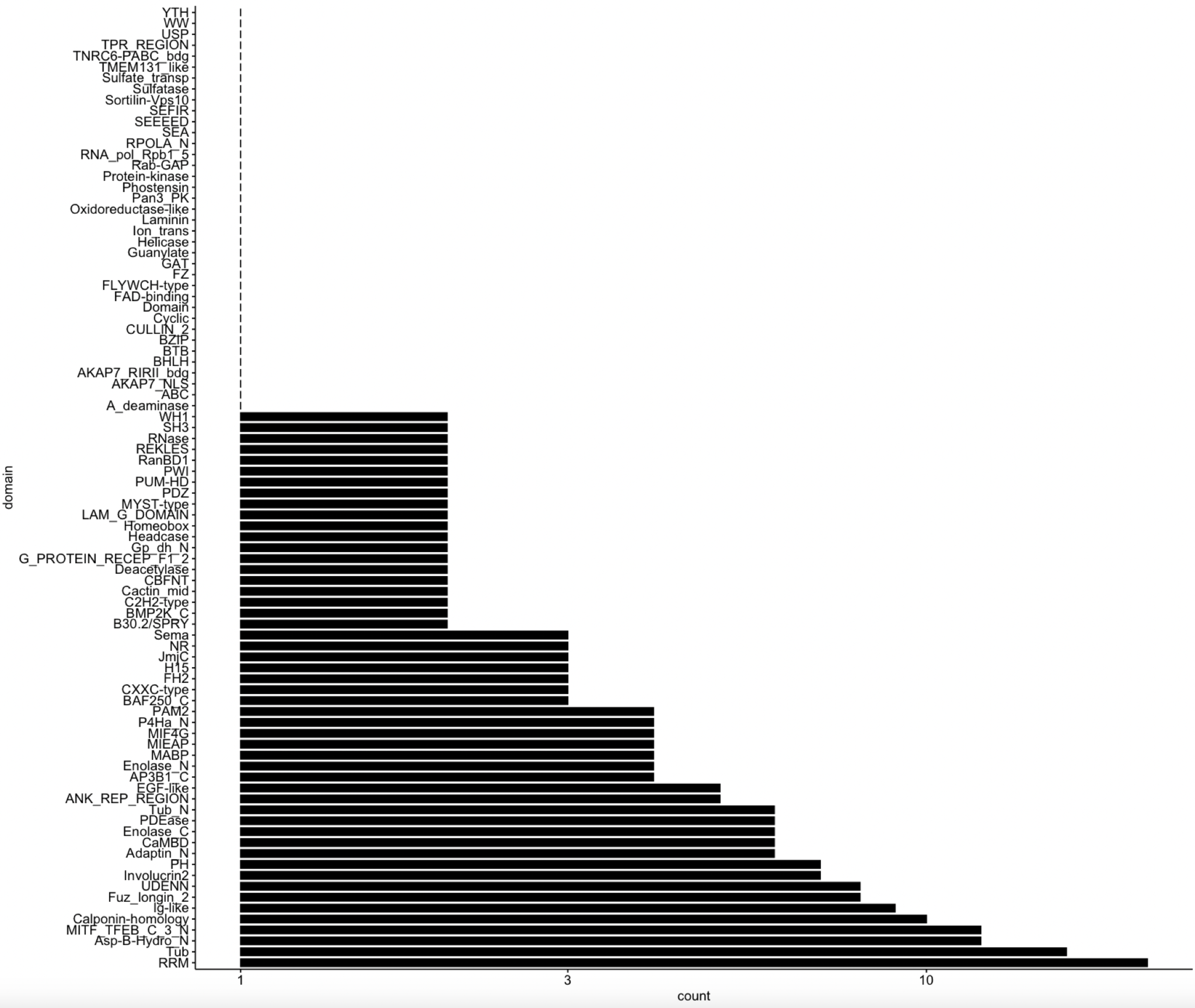
**

**Fig S1:** PTR extension alleles inside protein domains

Supplement: Supplementary file 1 — Additional file 1: Fig S1. PTR extension alleles inside protein domains. [file 12863_2022_1079_MOESM1_ESM.docx]
